# Characterization of Dysfunctional Lens Index and Opacity Grade in a Healthy Population

**DOI:** 10.3390/diagnostics12051167

**Published:** 2022-05-07

**Authors:** Elena Martínez-Plaza, Pedro Ruiz-Fortes, Roberto Soto-Negro, Carlos J. Hernández-Rodríguez, Ainhoa Molina-Martín, Alfonso Arias-Puente, David P. Piñero

**Affiliations:** 1 Group of Optics and Visual Perception, Department of Optics, Pharmacology and Anatomy, University of Alicante, 03690 Alicante, Spain; emartinezp@ioba.med.uva.es (E.M.-P.); hernandezrodriguezcj@gmail.com (C.J.H.-R.); ainhoa.molina@ua.es (A.M.-M.); 2 University of Valladolid, 47002 Valladolid, Spain; 3 Department of Ophthalmology, Vithas Medimar International Hospital, 03016 Alicante, Spain; ruizfp@vithas.es (P.R.-F.); sotonr@vithas.es (R.S.-N.); aap.oft@gmail.com (A.A.-P.)

**Keywords:** dysfunctional lens syndrome, cataract, dysfunctional lens index, high-order aberrations

## Abstract

This study enrolled 61 volunteers (102 eyes) classified into subjects < 50 years (group 1) and subjects ≥ 50 years (group 2). Dysfunctional Lens Index (DLI); opacity grade; pupil diameter; and corneal, internal, and ocular higher order aberrations (HOAs) were measured with the i-Trace system (Tracey Technologies). Mean DLI was 8.89 ± 2.00 and 6.71 ± 2.97 in groups 1 and 2, respectively, being significantly higher in group 1 in all and right eyes (both *p* < 0.001). DLI correlated significantly with age (Rho = −0.41, *p* < 0.001) and pupil diameter (Rho = 0.20, *p* = 0.043) for all eyes, and numerous internal and ocular root-mean square HOAs for right, left, and all eyes (Rho ≤ −0.25, *p* ≤ 0.001). Mean opacity grade was 1.21 ± 0.63 and 1.48 ± 1.15 in groups 1 and 2, respectively, with no significant differences between groups (*p* ≥ 0.29). Opacity grade significantly correlated with pupil diameter for right and all eyes (Rho ≤ 0.33, *p* ≤ 0.013), and with some ocular root-mean square HOAs for right and all eyes (Rho ≥ 0.23, *p* ≤ 0.020). DLI correlates with age and might be used complementary to other diagnostic measurements for assessing the dysfunctional lens syndrome. Both DLI and opacity grade maintain a relationship with pupil diameter and internal and ocular HOAs, supporting that the algorithms used by the device may be based, in part, on these parameters.

## 1. Introduction

Dysfunctional lens syndrome (DLS) has been defined as the age-related changes experienced by the crystalline lens [1]. The term involves three stages depending on the visual quality impairment and the age of the subject. Stage 1 is associated with a loss of accommodation that corresponds to presbyopia from 40 to 50 years old; stage 2 matches with an accommodation loss, early lens opacities, and increase in higher-order aberrations (HOAs) and light scatter—typically above 50 years and older; stage 3 is related to clinically significant cataract causing decreased functional vision in subjects equal or older than 65 years [2,3,4]. Thus, those subjects in stages 2 and 3 experience a visual quality deterioration. These ranges are frequently used in the clinical practice for both the assessment of the candidates to benefit from lens surgery and a better understanding of lens aging process. In both cases, the algorithms and automatic tools provided by the clinical platforms assist the clinician [5]. Specifically, objective measures of lens dysfunctionality and ocular scatter are required [2,6,7].

Dysfunctional lens index (DLI) and opacity grade are objective lens outcomes provided by the i-Trace Visual Function Analyzer (Tracey Technologies, Houston, TX, USA) in an attempt to quantify lens degradation. According to the manufacturer (Tracey VFA Visual Function Analyzer, operator’s manual), DLI values range from 0 to 10, and a lower value correlates to a more dysfunctional lens indicating an early cataract, while an increasing number correlates with a better lens performance. On the other hand, the opacity grade is based on statistical analysis of the overall opacity map, which considers the light energy from each of the first 128 beams that enter the pupil during the exam (Tracey VFA Visual Function Analyzer, operator’s manual). Although both parameters, DLI and opacity grade, could be considered to assist in the diagnosis and the optimal timing to proceed with a lens exchange [3,8], normal values in healthy population have not been described. Consequently, the aim of the present study was, first, to establish normal DLI and opacity grade values in a healthy population, and second, to analyze the relationship between DLI, opacity grade, pupil diameter, and ocular wavefront aberrations.

## 2. Materials and Methods

An observational study was performed in compliance with the tenets of the Declaration of Helsinki. The study was approved by the Ethics Committee of the University of Alicante and conducted at the Department of Ophthalmology of Vithas Medimar International Hospital (Alicante, Spain).

### 2.1. Sample

The present study included 102 eyes of 61 participants who understood and signed the informed consent. Inclusion criterion was subjects with a healthy eye without any previous ocular surgery or presence of any ocular pathology. Included patients were divided into 2 groups: subjects below 50 years old (group 1) and subjects equal to or above 50 years (group 2).

### 2.2. i-Trace System

**Figure 1:**
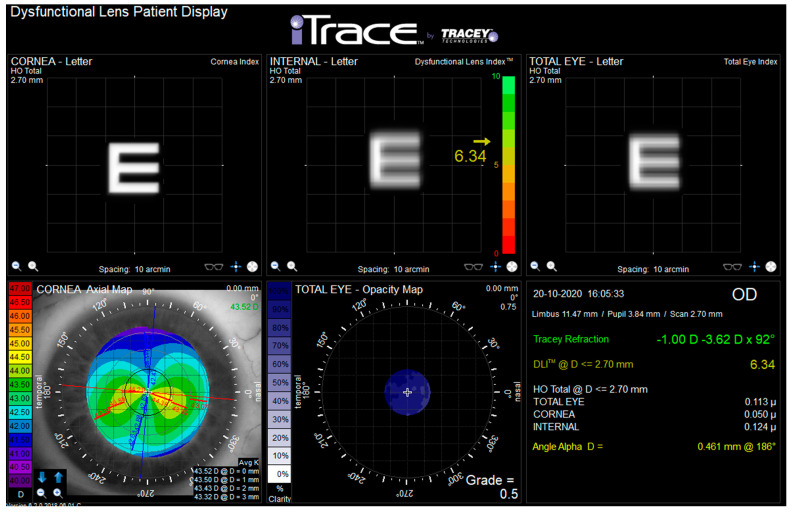
Dysfunctional lens patient display from i-Trace device. Dysfunctional lens index (DLI) is shown in yellow font in the top middle box. Opacity grade is shown in white font in the bottom middle box.

The i-Trace Visual Function Analyzer (Tracey Technologies, Houston, TX) combines a Placido disk-based corneal topography and a ray tracing aberrometer [9]. Measurements were performed in a dark room by the same experimented clinician (Piñero D.P.) during one study visit. After 2 min of dark adaptation [10], participants were conveniently positioned on the head-chin rest of the device. The ray tracing aberrometer of this system obtained a measurement of ocular aberrations, with a maximum area of analysis defined according to the level of pupil dilation achieved. After this, the Placido rings were projected onto the corneal tear film, and an autocaptured image was obtained. The i-Trace software then defined the ring edges and calculated the corneal curvature (simK steep, simK flat, simK axis, simK average, inferior–superior index, and corneal asphericity), corneal refractive power (sphere, cylinder, and axis), visual axes (angle kappa and angle alpha), and corneal wavefront data (corneal, internal, and ocular aberrations). With all this information, the software of the i-Trace system calculated the internal aberrations as well as the values of the DLI and opacity grade. Figure 1 represents the Dysfunctional Lens Patient Display shown by i-Trace.

From wavefront analysis, corneal, internal, and ocular HOAs were evaluated. The following HOAs for 3-mm and 6-mm pupil diameters were selected for study purposes: vertical coma (Z_3_^−1^), horizontal coma (Z_3_^1^), spherical aberration (Z_4_^0^), secondary spherical aberration (Z_6_^0^), primary (Z_3_^−1^ and Z_3_^1^) and secondary (Z_5_^−1^ and Z_5_^1^) coma root mean square (RMS), comalike (Z_3_^−1^, Z_3_^1^, Z_5_^−1^, and Z_5_^1^) RMS, spherical-like (Z_4_^0^ and Z_6_^0^) RMS, and total HOAs RMS (from 3rd to 5th order).

### 2.3. Statistical Analysis

The statistical analysis was performed using SPSS statistical package version 28.0.0 (IBM SPSS, Armonk, NY, USA). All eyes meeting the inclusion criteria were considered for the study; thus, both eyes of some subjects were included. Apart from analyzing all study eyes, statistical analysis was also performed by dividing the sample into right and left eyes.

Descriptive values provided for DLI and opacity grade were mean, standard deviation (SD), 95% confidence interval (CI), median, quartiles 1 and 3, minimum, and maximum. Differences between the two groups of the study were analyzed using the Student-T test for independent variables if the normality assumption was accomplished with the Shapiro–Wilk test; otherwise, the Mann–Whitney U test was used. Potential correlations of DLI and opacity grade with age and corneal, internal, and ocular aberrations were analyzed using the Spearman rank correlation coefficient.

*p*-values equal to or lower than 0.05 were considered statistically significant.

## 3. Results

A total of 102 eyes (56 right and 46 left eyes) of 61 subjects (32 females and 29 males) with a mean age of 46.7 ± 16.6 years were evaluated. Group 1 (<50 years), whose mean age was 34.6 ± 11.1 years, included 33 right eyes and 23 left eyes from 34 subjects; and group 2 (≥50 years), with a mean age of 62.0 ± 6.9 years, included 23 right eyes and 23 left eyes from 27 participants. Table 1 shows descriptive data from both groups. Mean values of corneal, internal, and ocular wavefront aberrations are presented in Table 2. One hundred and two eyes were included for wavefront analyses at 3 mm while 80 eyes (group 1: 30 right and 20 left eyes; group 2: 15 right and 15 left eyes) were included for analyses at 6 mm.

### 3.1. Dysfunctional Lens Index

Table 3 shows the descriptive values of DLI obtained for all, right, and left eyes of all participants and in each group. Group 1 showed significantly higher DLI values than group 2 for all eyes and right eyes (both *p* < 0.001), not achieving the difference between groups statistical significance for left eyes (*p* = 0.10).

Age significantly correlated with DLI for all eyes (Rho = −0.41, *p* < 0.001), for right eyes (Rho = −0.51, *p* < 0.001), and almost reached significance for left eyes (Rho = −0.26, *p* = 0.08). Gender did not have a significant influence on DLI for all eyes (*p* = 0.27), right eyes (*p* = 0.07), nor left eyes (*p* = 0.70).

### 3.2. Opacity Grade

Opacity grade descriptive data are presented in Table 3, classifying participants by groups and eyes. No significant differences were found for opacity grade between groups for all eyes, nor for right and left eyes (*p* ≥ 0.29).

Age did not correlate with opacity grade for any situation—all, right, and left eyes (*p* ≥ 0.61). Similarly, gender did not have a significant influence on opacity grade for all eyes or individual eyes (*p* ≥ 0.30).

### 3.3. Relationship between DLI and Opacity Grade

**Figure 2:**
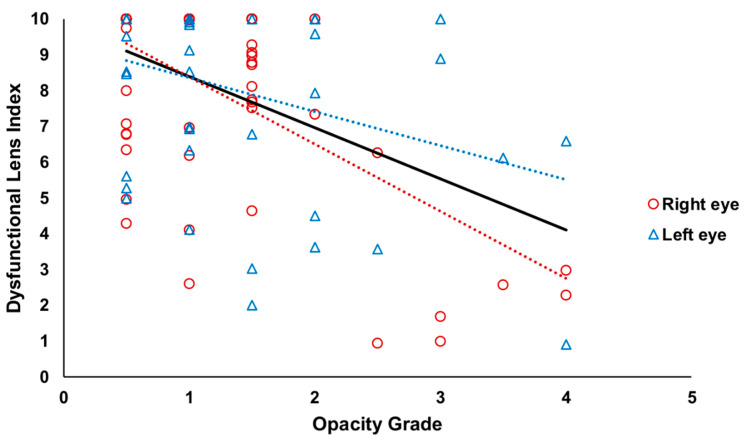
Scatter plot of the relationship between Dysfunctional Lens Index and Opacity Grade. Black, red, and blue lines indicate the tendency lines of all, right, and left eyes, respectively.

A significant correlation was found between both indexes, DLI and opacity grade, for all eyes (Rho = −0.33, *p* < 0.001) and right eyes (Rho = −0.38, *p* = 0.004). The relationship for left eyes did not reach statistical significance (Rho = −0.25, *p* = 0.10). Figure 2 presents the scatter plot representing this relationship between indexes.

### 3.4. Relationship of DLI with Pupil Diameter and Wavefront Aberrations

Pupil diameter has a significant relationship with DLI for all eyes (Rho = 0.20, *p* = 0.043), being close to significance for right eyes (Rho = 0.20, *p* = 0.07) and nonsignificant for left eyes (*p* = 0.31).

DLI correlated with some corneal aberrations for left eyes analyzed for 3- and 6-mm pupils, including primary spherical aberration (Z_4_^0^) (Rho ≥ 0.34, *p* ≤ 0.021), secondary spherical aberration (Z_6_^0^) and spherical-like RMS (Rho ≤ −0.30, *p* ≤ 0.041). Additionally, DLI correlated with spherical-like RMS for right eyes measured for a 3-mm pupil (Rho = −0.27, *p* = 0.048).

DLI was significantly correlated with internal primary spherical aberration for all eyes for a 3-mm pupil (Rho = −0.21, *p* = 0.031). Further, significant relationships were found between DLI and internal primary (Z_3_^−1^ and Z_3_^1^) and secondary (Z_5_^−1^ and Z_5_^1^) coma RMS, comalike (Z_3_^−1^, Z_3_^1^, Z_5_^−1^, and Z_5_^1^) RMS, spherical-like (Z_4_^0^ and Z_6_^0^) RMS, and total HOAs RMS for both pupil diameters in right, left, and all eyes (Rho ≤ −0.37, *p* < 0.001), except for spherical-like RMS measured for a 3-mm pupil in the left eye (Rho = −0.26, *p* = 0.09).

Significant correlations were found between DLI and ocular primary spherical aberration measured for a 3-mm pupil for right and all eyes (Rho = −0.32, *p* = 0.017; Rho = 0.33, *p* = 0.017, respectively). In addition, significant relationships were found between DLI and ocular primary (Z_3_^−1^ and Z_3_^1^) and secondary (Z_5_^−1^ and Z_5_^1^) coma RMS, comalike (Z_3_^−1^, Z_3_^1^, Z_5_^−1^ and Z_5_^1^) RMS, spherical-like (Z_4_^0^ and Z_6_^0^) RMS, and total HOAs RMS for both pupil diameters in right, left, and all eyes (Rho ≤ −0.25, *p* ≤ 0.001).

### 3.5. Relationship of Opacity Grade with Pupil Diameter and Wavefront Aberrations

Pupil diameter has a significant influence on opacity grade for right and all eyes (Rho = 0.28, *p* = 0.004 and Rho = 0.33, *p* = 0.013, respectively), not reaching significance for left eyes (*p* = 0.23).

A negative correlation was found between opacity grade and corneal HOAs RMS in left eyes measured for a 3-mm pupil (Rho = −0.30, *p* = 0.042). Opacity grade positively correlated with internal spherical-like RMS in right and all eyes measured for both 3 and 6-mm pupils (Rho ≥ 0.25, *p* ≤ 0.027). Regarding ocular aberrations, opacity grade was correlated with horizontal coma (Z_3_^1^) for right eyes measured for a 6-mm pupil (Rho = 0.39, *p* = 0.007), with primary coma RMS and comalike RMS for right eyes and all eyes measured for 3 and 6-mm pupils (Rho ≥ 0.27, *p* ≤ 0.005), with spherical-like RMS for right eye for 6-mm pupil and all eyes for 3-mm pupil (Rho ≥ 0.23, *p* ≤ 0.020), and with HOAs RMS for right and all eyes measured for a 6-mm pupil (Rho ≥ 0.28, *p* ≤ 0.011).

## 4. Discussion

Nowadays, the use of automatic devices and algorithms applied to the measurement of clinical parameters is very extended. In the ophthalmology field, clinical platforms assist clinicians in the diagnosis and follow-up of pathologies, the improvement of surgical protocols, and the prevention of undesirable events [11,12,13]. In this regard, DLS assessment is gaining relevance to better adapt the adequate timing for the lens exchange procedure [4,14]. Since DLI and, likely, opacity grade could be useful in DLS consideration [3,8], the current study describes normal DLI and opacity grade values in a healthy population (<50 years and ≥50 years) and analyzes the relationship between DLI, opacity grade, and wavefront aberrations obtained with the i-Trace device. We found that mean DLI values are near to 8 points, being significantly lower in the older group, while opacity grade values were similar in both groups, near to 1 point. Finally, both indexes presented a weak-to-moderate although statistically significant correlation, and both correlated as well with ocular and internal HOAs. These findings are valuable for the objective assessment of DLS by clinicians and researchers.

In the present study, approximately 75% of the subjects in the younger group showed DLI values between 8 and 10 points; although, on the contrary, it is remarkable that a lower percentage of subjects obtained lower values. Regarding subjects older than 50 years, the mean DLI value was significantly lower, by more than 2 points, than in the younger group. This outcome was to be expected, considering that a lower DLI value is said to be associated with a more dysfunctional lens. Although little literature has been reported about DLI, similar mean values have been previously reported for slightly older populations [8,15,16,17]. Besides, we also found an inverse correlation between DLI and age, supporting the findings abovementioned. Therefore, DLI could be considered as a useful indicator for DLS assessment in the clinical practice, although the variability observed implies the necessity of combining with other diagnostic measurements. Similarly, additional factors, such as lens dimensions and anterior chamber assessment, are required for considering any lens exchange procedure [18].

Analyzing the relationship of DLI with other parameters, DLI positively correlated with pupil diameter for all eyes. The correlations were not significant analyzing each eye individually, although the lack of significance (almost reached for the right eye) could be the consequence of the lower sample size achieved by dividing the sample evaluated into right and left eyes. Additionally, DLI showed a limited relationship with corneal wavefront aberrations and a weak-to-moderate but statistically significant inverse correlation with internal and ocular HOA RMS for both pupil diameters, 3 mm and 6 mm. These outcomes indicate that lower DLI values are associated with higher RMS HOAs, supported by the fact that higher HOAs appear in visual-deteriorated optical systems [19,20,21]. Altogether, these findings corroborate that, despite not knowing the exact algorithm used by the i-Trace to calculate DLI, this index may be, in part, sustainable in pupil size and several HOAs.

The opacity grade values ranged between 0.5 and 4 for all subjects. Despite that opacity grade could be understood as an indicator of lens opacification, surprisingly, no differences were found in the mean value between groups; in fact, the opacity grade did not correlate with age. As far as we know, no previous studies have reported the values obtained for this index with the i-Trace. Besides, there is a gap in the knowledge about the algorithm used by the device to calculate the opacity grade. However, we found some limited associations with spherical and coma aberrations (internal and ocular wavefront data) which, indeed, are widely associated with the presence of cataract [20,22,23]. In addition, an inverse significant correlation was also found between DLI and opacity grade indicating that subjects with higher lens opacification (higher opacity grade) present more dysfunctional lenses (lower DLI). Thus, based on our outcomes, opacity grade appears to be an uncertain measurement for discerning between young individuals and subjects in stages 2–3 of DLS.

In the current study, subjects were divided into young individuals and stages 2–3 of DLS, considering the accepted definition of DLS [2,3,4]. However, the absence of a procedure to evaluate the sclerotic grade of the crystalline lens of the patients evaluated could be considered as a limitation. Besides, the lack of biometric data associated with age-related changes in the anterior segment could also be considered as a drawback. In addition, the sample size could be considered modest; thus, future studies with larger sample sizes are needed. Finally, additional associations with quality of vision parameters, such as quality of vision questionnaires or contrast sensitivity function, between others, could be of interest from a clinical viewpoint.

## 5. Conclusions

In conclusion, the normal DLI and opacity grade values in a healthy population below 50 years and in subjects in stages 2–3 of DLS are provided, which could be used as a reference for an adequate interpretation of these i-Trace indexes by clinicians and researchers. In addition, DLI has been corroborated as a valuable and objective tool, complementary to other diagnostic measurements, for surgeons to assess DLS in the daily clinical practice. On the other hand, opacity grade appears not to be an accurate indicator for stages 2–3 of DLS. Finally, both indexes maintain a relationship with pupil diameter and internal and ocular HOAs, supporting that the algorithms used by the device may be based, in part, on these parameters.

## Figures and Tables

**Table 1:** Ocular parameters and statistical differences between subjects below 50 years (Group 1) and subjects equal or above 50 years (Group 2).

	Right Eyes			Left Eyes		
Parameters	Group 1	Group 2	*p*-Value	Group 1	Group 2	*p*-Value
Sphere (D)	−2.90 ± 3.05	−0.09 ± 2.65	<0.001	−3.51 ± 3.97	−0.04 ± 1.94	<0.001
Cylinder (D)	−1.18 ± 0.96	−1.27 ± 0.78	0.33	−1.08 ± 1.08	−1.32 ± 0.75	0.09
Axis (mm)	93.70 ± 63.19	113.52 ± 33.67	0.38	90.43 ± 62.61	90.09 ± 42.37	0.82
Angle kappa distance (mm)	0.30 ± 0.16	0.40 ± 0.25	0.14	0.41 ± 0.15	0.31 ± 0.11	0.01
Angle kappa (degrees)	205.48 ± 43.33	178.43 ± 55.68	0.06	192.87 ± 153.26	242.78 ± 135.82	0.04
Angle alpha distance (mm)	0.41 ± 0.12	0.43 ± 0.11	0.57	0.31 ± 0.12	0.33 ± 0.13	0.96
Angle alpha (degrees)	183.42 ± 25.72	186.26 ± 7.42	0.71	321.17 ± 95.91	206.09 ± 170.55	0.007
Pupil diameter (mm)	5.83 ± 1.17	4.86 ± 0.82	<0.001	5.52 ± 1.26	4.71 ± 1.09	0.012
Limbal diameter (mm)	11.49 ± 0.39	11.53 ± 0.38	0.74	11.55 ± 0.43	11.52 ± 0.44	0.69
Q value	−0.30 ± 0.35	−0.23 ± 0.43	0.12	−0.23 ± 0.12	−0.08 ± 0.55	0.12
Simk steep (D)	43.50 ± 2.02	42.74 ± 2.09	0.18	43.23 ± 1.86	42.90 ± 1.86	0.85
Simk flat (D)	42.30 ± 2.12	41.79 ± 1.94	0.28	42.23 ± 1.95	42.00 ± 1.76	0.53
Simk flat axis (D)	89.79 ± 78.34	114.13 ± 52.39	0.54	83.83 ± 74.71	95.17 ± 58.76	0.50
Simk average (D)	42.89 ± 2.04	42.26 ± 2.00	0.25	42.72 ± 1.86	42.44 ± 1.77	0.65
Inferior-superior difference (D)	0.14 ± 0.45	0.26 ± 0.87	0.55	0.29 ± 0.48	0.42 ± 1.13	0.64

D: diopters. Data are presented as mean ± standard deviation. *p*-value indicates the comparison between groups.

**Table 2:** Mean values of corneal, internal, and ocular wavefront aberrations in all, right, and left eyes for 3-mm and 6-mm pupil diameters.

		3 mm			6 mm		
Parameters (µm)		Corneal	Internal	Ocular	Corneal	Internal	Ocular
Z_3_^−1^	AE	−0.002 ± 0.015	−0.006 ± 0.039	−0.007 ± 0.039	−0.006 ± 0.038	−0.003 ± 0.067	−0.010 ± 0.074
	RE	−0.03 × 10^−1^ ± 0.015	−0.005 ± 0.027	−0.005 ± 0.027	−0.04 × 10^−1^ ± 0.041	−0.09 × 10^−2^ ± 0.063	−0.007 ± 0.073
	LE	−0.003 ± 0.015	−0.008 ± 0.050	−0.011 ± 0.051	−0.009 ± 0.036	−0.005 ± 0.070	−0.013 ± 0.076
Z_3_^1^	AE	0.01 × 10^−2^ ± 0.010	0.006 ± 0.030	0.006 ± 0.030	0.02 × 10^−2^ ± 0.031	0.006 ± 0.055	0.004 ± 0.061
	RE	−0.003 ± 0.007	0.008 ± 0.034	0.006 ± 0.034	−0.007 ± 0.020	0.013 ± 0.060	0.006 ± 0.066
	LE	0.003 ± 0.013	0.004 ± 0.025	0.007 ± 0.027	0.009 ± 0.039	−0.001 ± 0.047	0.001 ± 0.057
Z_4_^0^	AE	0.003 ± 0.003	0.003 ± 0.017	0.005 ± 0.017	0.013 ± 0.011	0.010 ± 0.073	0.027 ± 0.081
	RE	0.003 ± 0.003	0.004 ± 0.021	0.007 ± 0.021	0.013 ± 0.014	0.012 ± 0.076	0.028 ± 0.084
	LE	0.003 ± 0.002	0.05 × 10^−2^ ± 0.011	0.003 ± 0.011	0.013 ± 0.008	0.009 ± 0.070	0.027 ± 0.079
Z_6_^0^	AE	0.000 ± 0.000	−0.04 × 10^−2^ ± 0.003	−0.04 × 10^−2^ ± 0.003	−0.04 × 10^−3^ ± 0.10 × 10^−2^	−0.001 ± 0.011	−0.08 × 10^−2^ ± 0.011
	RE	−0.001 × 10^−3^ ± 0.001	−0.04 × 10^−2^ ± 0.003	−0.04 × 10^−2^ ± 0.001	−0.01 × 10^−2^ ± 0.001	−0.003 ± 0.012	−0.003 ± 0.013
	LE	−0.001 × 10^−3^ ± 0.07 × 10^−3^	0.04 × 10^−2^ ± 0.003	−0.04 × 10^−2^ ± 0.003	0.08 × 10^−3^ ± 0.07 × 10^−1^	0.04 × 10^−2^ ± 0.009	0.002 ± 0.008
Primary Coma RMS	AE	0.013 ± 0.012	0.037 ± 0.046	0.032 ± 0.039	0.036 ± 0.034	0.064 ± 0.069	0.066 ± 0.071
	RE	0.013 ± 0.010	0.037 ± 0.048	0.030 ± 0.032	0.036 ± 0.029	0.065 ± 0.079	0.064 ± 0.074
	LE	0.013 ± 0.015	0.036 ± 0.045	0.036 ± 0.047	0.038 ± 0.039	0.063 ± 0.055	0.068 ± 0.068
Secondary Coma RMS	AE	0.03 × 10^−2^ ± 0.002 × 10^−2^	0.005 ± 0.007	0.005 ± 0.007	0.001 ± 0.002	0.017 ± 0.025	0.016 ± 0.027
	RE	0.03 × 10^−2^ ± 0.02 × 10^−2^	0.005 ± 0.006	0.005 ± 0.006	0.002 ± 0.003	0.017 ± 0.022	0.016 ± 0.023
	LE	0. 03 × 10^−2^ ± 0.01 × 10^−2^	0.005 ± 0.008	0.005 ± 0.008	0.002 ± 0.001	0.017 ± 0.029	0.017 ± 0.031
Coma-like RMS	AE	0.013 ± 0.012	0.038 ± 0.047	0.033 ± 0.040	0.037 ± 0.034	0.068 ± 0.071	0.070 ± 0.074
	RE	0.013 ± 0.010	0.038 ± 0.048	0.031 ± 0.032	0.036 ± 0.028	0.047 ± 0.080	0.068 ± 0.076
	LE	0.013 ± 0.015	0.036 ± 0.045	0.037 ± 0.047	0.038 ± 0.039	0.067 ± 0.060	0.072 ± 0.072
Spherical-like RMS	AE	0.004 ± 0.004	0.009 ± 0.015	0.010 ± 0.015	0.014 ± 0.011	0.029 ± 0.068	0.036 ± 0.079
	RE	0.004 ± 0.004	0.010 ± 0.019	0.011 ± 0.019	0.014 ± 0.013	0.031 ± 0.071	0.037 ± 0.081
	LE	0.004 ± 0.002	0.008 ± 0.008	0.008 ± 0.008	0.013 ± 0.008	0.027 ± 0.065	0.034 ± 0.077
HOAs RMS	AE	0.021 ± 0.020	0.057 ± 0.061	0.057 ± 0.059	0.063 ± 0.057	0.117 ± 0.115	0.121 ± 0.122
	RE	0.022 ± 0.021	0.061 ± 0.067	0.058 ± 0.063	0.065 ± 0.065	0.126 ± 0.131	0.126 ± 0.135
	LE	0.021 ± 0.018	0.053 ± 0.053	0.055 ± 0.053	0.061 ± 0.047	0.107 ± 0.092	0.115 ± 0.104

Data are presented as mean ± standard deviation. AE—all eyes; RE—right eye; LE—left eye.

**Table 3:** Dysfunctional lens index and opacity grade descriptive data.

			Mean ± SD	95% CI	Median [IQR]	Range
Dysfunctional Lens Index	All subjects	AE	7.91 ± 2.70	7.38/8.44	9.40 [6.34–10.00]	0.90/10.00
		RE	7.84 ± 2.80	7.09/8.59	9.17 [6.45–10.00]	0.93/10.00
		LE	7.99 ± 2.61	7.22/8.77	9.55 [6.27–10.00]	0.90/10.00
	Group 1	AE	8.89 ± 2.00	8.36/9.43	10.00 [8.22–10.00]	2.00/10.00
		RE	9.18 ± 1.42	8.67/9.68	10.00 [8.42–10.00]	4.63/10.00
		LE	8.49 ± 2.60	7.36/9.62	10.00 [7.93–10.00]	2.00/10.00
	Group 2	AE	6.71 ± 2.97	5.83/7.60	6.95 [4.24–9.93]	0.90/10.00
		RE	5.93 ± 3.19	4.55/7.31	6.34 [2.60–8.98]	0.93/10.00
		LE	7.49 ± 2.57	6.38/8.61	8.46 [5.61–10.00]	0.90/10.00
Opacity Grade	All subjects	AE	1.33 ± 0.91	1.16/1.51	1.00 [0.50–1.50]	0.50/4.00
		RE	1.29 ± 0.89	1.05/1.52	1.00 [0.50–1.50]	0.50/4.00
		LE	1.39 ± 0.94	1.11/1.67	1.00 [0.50–2.00]	0.50/4.00
	Group 1	AE	1.21 ± 0.63	1.05/1.38	1.00 [0.50–1.50]	0.50/3.00
		RE	1.09 ± 0.57	0.89/1.29	1.00 [0.50–1.50]	0.50/2.50
		LE	1.39 ± 0.69	1.09/1.69	1.00 [1.00–2.00]	0.50/3.00
	Group 2	AE	1.48 ± 1.15	1.14/1.82	1.00 [0.50–2.00]	0.50/4.00
		RE	1.57 ± 1.17	1.06/2.07	1.00 [0.50–2.50]	0.50/4.00
		LE	1.39 ± 1.15	0.90/1.89	1.00 [0.50–2.00]	0.50/4.00

Data are presented as mean ± standard deviation. AE—all eyes; RE—right eye; LE—left eye.

## Data Availability

Data available on request from the authors.
